# How organizational learning is associated with patient rights: a qualitative content analysis

**DOI:** 10.3402/gha.v9.30939

**Published:** 2016-07-26

**Authors:** Shahin Heidari, Nahid Dehghan Nayeri, Ali Ravari, Sakineh Sabzevari

**Affiliations:** 1 Nursing Research Center, Razi Faculty of Nursing and Midwifery, Kerman University of Medical Sciences, kerman, Iran; 2 Nursing and Midwifery Care Research Center, School of Nursing and Midwifery, Tehran University of Medical Sciences, Tehran, Iran; 3 Geriatric Care Research Center, School of Nursing and Midwifery, Rafsanjan University of Medical Sciences, Rafsanjan, Iran; 4 School of Nursing and Midwifery, Kerman University of Medical Sciences, Kerman, Iran

**Keywords:** organizational learning, patient rights, patient satisfaction

## Abstract

**Background:**

Nowadays, patient rights, particularly receiving favorable health care based on modern knowledge, informed consent, and privacy, are important issues in health care delivery systems. Organizational learning is considered an important factor influencing health care quality and patient rights. However, there is little evidence regarding this issue.

**Objective:**

The present study was conducted to explore the role of organizational learning in patient rights from clinical nurses’ viewpoint.

**Design:**

This qualitative study was conducted through conventional content analysis. In total, 18 nurses who met the inclusion criteria participated in this study through purposive sampling with maximum variation. Data were gathered through 20 in-depth, semi-structured interviews, which continued until data saturation was achieved. Data collection also included constant and simultaneous comparative analyses.

**Results:**

Data analysis led to four major themes: conservation of patient safety, providing favorable care, being the patient's advocate, and informing the patients. All the participants believed that organizational learning could play a vital role in respecting patient rights and interests.

**Conclusions:**

Participants believed that their efforts to conduct organizational learning, tried to improve respecting the patient rights via conservation of patient safety, trying to improve quality of care, being an advocate, and informing the patient. It would be appreciable if nursing managers honored the commitment of the nurses for learning, highlight their role as defenders of patient rights, and encourage them to initiate organizational learning.

## Introduction

Patient rights are the rules of communication between patients, health professionals, and health services (1). The World Medical Association (WMA) published the first international document about patient rights in 1981, and new notifications were published by the World Health Organization (WHO) and WMA (2). Issues concerning patient rights include respecting the patient as a human being, providing favorable care, offering information related to medical processes, and privacy (3).

According to the Constitution of Iran, paying attention to human dignity is one of the building blocks of the Islamic Republic system, and offering health services must be equitable and should be based on respecting and preserving the human dignity of the patients. Hospitalized patients become a part of the most vulnerable people in the society because they lose some capabilities; therefore, it seems necessary to have some strategies to preserve their rights. Developing patient rights, which was announced to all medical sciences universities throughout the country in December 2009 (4), is one of the activities conducted in this context. This bill stipulates that receiving favorable health services is the patient's right, and offering health services should be based on modern knowledge and highlight patient's interests (5). Organizational learning is considered as a way to achieve providing favorable care through a set of organizational practices, including learning knowledge, distributing and interpreting the information, and conscious or unconscious memorizing of the information (6). Organizational learning is a cumulative process in which personal and group experiences are transferred to organizational routines, processes, and structures and in turn affect the future learning of organization members (7). Crossan's definition of organizational learning involves four processes including *intuiting* (the process of developing new insights and ideas), *interpreting* (explaining the insights to him/herself and to others), *integrating* (shared understanding among individuals and groups is achieved), and finally *institutionalizing* (shared understanding is implemented in systems, structures, procedures, rules, and strategies) (8). Finally, Argot defined it as an ongoing cycle in which work experiences produce knowledge in interaction with context. The knowledge transmitted from organization to environment, changes the context of the organization and affects the future learning (9).

A review of various studies shows that the consequences of organizational learning are not adequately discussed. Perhaps different definitions of organizational learning affect the consequences of its definition, as few articles offered experimental evidence concerning appropriate organizational consequences or results. A systematic review found no evidence to support or reject the relationship between organizational learning and practices. However, a range of consequences, including individual's behavioral changes (better performance of responsibilities), changes in the system (logistic, for instance), and changes in organizational performances (such as financial consequences), are reported in some articles (10). Other articles report that the consequences of organizational learning includes its influence on job satisfaction, the ability to accept and face changes and challenges, employee's organizational commitment (11, 12), and their professional competence promotion (13). Studies conducted in Iran also emphasize a strong correlation between the elements of organizational learning and empowerment (14–17). However, the association between organizational learning and patient's rights is not mentioned in these articles. Although patients may be unaware of their rights (18), preserving patients’ rights can improve patient safety (19) and patient satisfaction (20).

As mentioned earlier, there is little evidence showing the influence of organizational learning on nurses’ activities, particularly in the scope of patient rights. Existing studies have separately discussed one of the issues related to organizational learning or patient rights, but their relationships have been ignored. Moreover, these studies are quantitative and offer no clear picture of these two concepts and their relationship. Because of the limitations of the existing studies concerning this issue, it seemed necessary to conduct a qualitative study for an in-depth analysis in these dimensions. It must be noted that the provisions of this bill in Iran are rooted in Islamic learning, and the religious beliefs built upon respecting noble human dignity and patient rights are considered not only as a civil right but also as natural patient rights whose sustenance by health care employees is an ethical and legal responsibility. As a result, paying attention to rights bears high sensitivity. *The present study was conducted to explore the role of organizational learning in patient rights from clinical nurses’ viewpoint*.

## Theoretical framework

The theoretical framework of this study is based on the Crossan et al. (8) model of organizational learning which often used in academic contexts. The value of the proposal lies in its integration of three levels of learning, namely individual, group, and organizational learning; and of two routes of learning: from the individual to the organization and from the organization to the individual.

Individual learning needs a transference process of knowledge among people, with the purpose of institutionalization. This model identifies four processes of learning: intuiting, interpreting, integrating, and institutionalizing. The first process, intuiting, takes place at the individual level. The interpretation process occurs at the individual and group levels. It is defined as ‘the explaining of an insight or idea to one's self and to others’. Individuals think about their intuitions and share them with others, thus transferring them to individual and collective interpretation.

The integrating process is defined as ‘the process of developing shared understanding among individuals and of taking coordinated action through mutual adjustment. Dialogue and joint action are crucial to the development of shared understanding’.

The fourth concept, institutionalizing, ‘is the process of ensuring that routinized actions occur. This is the process of embedding learning that has occurred by individuals and groups into the organization and it includes systems, structures, procedures, and strategy’ (8).

## Methods

In this qualitative content analysis, 18 nurses working in the clinical wards of three hospitals affiliated with Kerman University of Medical Sciences, who met the study inclusion criteria, were selected through purposive sampling with a maximum variation. This sampling method is often used in qualitative studies for its advantages and its ability to properly clarify the phenomenon and identify the primary patterns involved in that variation (21). Having a bachelor's or a higher degree in nursing; having at least 1 year of clinical work experience; being currently employed in clinical wards; being physically, mentally, and cognitively healthy; and being able to communicate and share experiences were the inclusion criteria in the present study. *The first interviews were done by a key informant participant, who had enriched experiences regarding various roles of a clinical nurse. The sampling process continued by choosing* participants including both males and females, with different job positions and levels of education, different age groups, and different work experiences for obtaining accurate data and gaining a deep understanding of the different dimensions of the phenomenon. According to Marshall and Martin (22) an appropriate sample size for a qualitative study is one that adequately answers the research question. Therefore, sampling continued until no new categories, themes, or explanations emerged (data saturation).

Data were collected using 20 in-depth semi-structured interviews to extract complicated answers containing a large amount of information. This enabled the researcher to perceive how participants think and feel, what experiences they have, and how they perceive the world. The interview began with general and personal questions as well as open-ended ones based on the study objectives. For example, ‘Can you talk about the role of organizational learning on your performance? Can you talk about experiences of organizational learning outcomes on patients? Do you have any experience regarding organizational learning's role on respecting patient rights?’ The interview then continued with exploratory questions to clarify the concept and get more in-depth information. For example, ‘What happened in those situations? What did you do?’ Based on the emerging data and the interview progression, further questions were asked. The process of data collection continued until data saturation occurred, i.e. when all the concepts under study were clearly defined and further interviews did not generate new categories. Interviews lasted for 45–100 min (with a mean duration of 65 min) and were recorded using a digital recorder. The participants were asked to determine the place and time of interview according to their preferences. This allowed maximum concentration and prevented disruption of their thoughts by intervening factors.

Data extracted from 20 in-depth, semi-structured interviews with 18 nurses, were analyzed through conventional content analysis. The researcher listened to the interviews several times immediately after they were conducted and then transcribed them verbatim. For a better immersion in the data and to obtain a general idea of data, the analysis began with frequent readings of the entire text. At the first meeting, units were determined from participant statements in the interviews and other data were obtained from observations and field notes and labeled as codes. Then codes were arranged and classified into primary categories according to their similarities and differences with the cooperation and agreement of the research team. Subcategories were formed with similar events and outcomes, and the primary categories were then set. This subjective procedure continued until the themes were extracted (23). The researcher used MAXQDA-10 to facilitate data analysis, categorization, constant comparisons, and quotation retrieval.

The rigor of the data was determined using Lincoln and Guba's evaluative criteria, including credibility, dependability, transferability, and conformability (24). Long-term involvement, the researcher's extensive contact with the data, triangulation in data collection, constant comparison, revision of the coding and categorizations by the research team, and peer check were used to ensure credibility of the data. Dependability of data was assessed through member check by colleagues and participants.

Detailed description of the research process and results was the approach to ensure data transferability.

### Research ethics

The study was approved by the Research Council of the School of Nursing and the Ethics Committee of Kerman University of Medical Sciences under the ethical compliance code of K/93/154 and the approval code of 93/305. The researcher introduced herself and explained the research objectives to eligible nurses and then invited them to participate in the study. Written informed consent was obtained for participation in the study and recording interviews. The names and any other identification clues were removed from both the recorded interviews and transcriptions. Participants were ensured that they could withdraw from the study whenever they wanted. Ethical considerations were respected during all the processes of the study, including data collection (recording and transcribing the interviews), data analysis, and dissemination of results.

## Results

In total, 20 interviews were conducted with 18 nurses, including 13 women and 5 men, comprising nursing experts (11 individuals), MSc (5 individuals), and nursing PhD candidates (2 individuals), with a mean age of 34.8 years and 12.3 years of job experience. Interviews were conducted at the participant's workplace (14 participants), researcher's workplace (2 participants), and participants’ homes (2 participants). Data analysis revealed four major themes: preserving patient safety, providing favorable care, being the patient's advocate, and informing the patient (Table 1).

**Table 1:** Major themes and subthemes of study

Theme	Subtheme
Preserving patient safety	Sensitivity to errors Problem-detecting processes Learning from errors
Providing favorable care	Providing suggestions Decreasing or eliminating identifiable threats
Being patient's advocate	Learning for secure care Hazards of insufficient knowledge
Informing the patients	–

### Preserving patient safety

Searching for evidence and exchanging information among colleagues affected nurses’ activities and made them sensitively preserve patient safety through planning problem-detecting processes and learning from errors. In other words, organizational learning was consistent with preserving patient safety.

#### Sensitivity to errors

Nurses were sensitive to performing errors. By referring to evidence and offering the information to others, they tried to prevent performing any errors and hurting the patients in every possible way. One of the nurses said: ‘In dialysis unit, heparin is injected to the patients at first. I checked and found it scientifically wrong. Heparin lasts for 2 h, while dialysis lasts 4 h. My patient had blood circulation without heparin for 2 h. There was also clots in tubes. I said it clearly to all the personnel and the supervisor. I pointed the clots to them. Do you know why? Because I don't want my patient to be hurt’ (Participant No. 11).

#### Problem-identification processes

Detailed observation, monitoring the ward's events, and developing special instructions to highlight the existing defects constitute the dimensions of the problem-identification process. For instance, one of the participants said: ‘We asked the evening and night supervisor to write down patient record problems and generally all the problems of the ward in a notebook. By reviewing that notebook, we quickly understand the problems. Then, we think how to resolve the problems’ (Participant No. 1).

#### Learning from errors

Learning from errors is a basic part of organizational learning that plays an important role in improving patient safety. When nurses learned about an error that threatened a patient's life, they analyzed the problem and the possible resolutions and prevented its recurrence. The following are instances of learning from errors: ‘Once, one pipette of a dialysis machine was accidentally in acid. If it were connected to the patient, he would die. We wondered what to do, and mentioned the solutions. Finally, we decided to take the pipette back and fasten it with a wire to the back of the machine. It's a simple method, but it practically saves the life of the patient’ (Participant No. 6).

Another participant states her experience about learning from errors, which saved a patient from a dangerous transfusion reaction: ‘Once, a blood sample that was supposed to be sent for blood reservation was sent wrongly. When they came to inject the blood, they knew that the blood didn't match the patient. The patient survived a big threat. We decided what to do to prevent such errors. One nurse suggested having one pipe stand above each bed. Every day we put two tagged pipes there for the occasions we're in hurry and prevented such errors’ (Participant No. 12).

### Providing favorable care

Using collaborative learning method and teamwork, which were taken from organizational learning rules, nurses offer suggestions to decrease or eliminate identifiable threats whose outcome improves the quality of care.

#### Providing suggestions

Nurses were sensitive to problems in the ward. Rather, they offer suggestions to eliminate them through using collaborative organizational learning approach and teamwork. One nurse described teamwork in their setting as an interesting experience: ‘We had a notebook in the ward. We wrote all the problems in it. Then, we discussed it with our colleagues. Each time a problem was resolved, we enthusiastically went to resolve other ones. It was really interesting’.

She also stated a suggestion that has a valuable effect on patient care*:* ‘It's really difficult and dangerous to move patients under ventilators to dialysis ward. We suggested a dialysis machine inserted in that unit. Also we accept the responsibility for staff education to enable them to perform dialysis for these patients. So, we prevent some dangers due to transferring a patient with a bad clinical condition’ (Participant No. 3).

It is noteworthy that nurses tried to apply some of their suggestions not through the official channels or with a hope of support; rather, they occasionally applied them at their personal costs. For example one participant said: ‘Patients asked a lot of questions, and we had to answer them with the help of our colleagues. We wrote down all the possible questions a patient might have with their answers. We published it as a booklet. We extracted some pamphlets out of this booklet and distributed them among the patients. Such works were out-of-hospital activities, but we did them with the personal money each month we save for our patients. If our suggestions fall in the hold of official hierarchy, it was unclear when they're done’ (Participant No. 4).

There were instances where despite nurses’ efforts, they could not make their suggestions applicable because they did not receive any support from the health system. One of the participants described her experience as follows: ‘Unfortunately, we don't have any centers to refer our dialysis patients to. The patient paid much money and gains nothing. We suggested establishing a counseling center to inform them. We asked to refer patients with renal failure to this center. They (officials) pretend to support us, but practically none is achieved’ (Participant No. 4).

#### Decreasing or eliminating identifiable threats

Using the most economical and cheapest method, nurses took actions in decreasing or eliminating risks. For example, one participant said about control of infections in dialysis patients using some simple measures: ‘Once, we observed that AV fistula site infections are increasing. We prepare sterile perforated sheets. When we compared the amounts of infections before and after our action, we knew that the infection have thoroughly vanished. It was that effective’ (Participant No. 7).

Another nurse stated an innovation aimed at decreasing threats during batching in the ICU patients: ‘We were innovators. Most ICU patients were bathed on the bed. We scrutinized and found out that we could carefully take patients with ventilators to the bath. It was a new method used nowhere before. We were the only unit to do that’ (Participant No. 9).

### Being advocates

Learning for secure care and the hazards of nurse's insufficient knowledge are the major subthemes.

#### Learning for secure care

Receiving secure care is a part of patient's bill of rights, and offering such services must prioritize the patients’ interests and must be based on modern knowledge. One of the most important incentives of the nurses to learn modern knowledge was to prioritize and preserve the patient's interests. The following experience is an example for offering favorable care: ‘The doctor prescribed 9 million units of Colistin (the strongest antibiotic) for the patient …. I myself translated its brochure; it said that maximum dose is 6 million units per day. I told it to the doctor and he called it nonsense. I spent a week for that. But I continue my manner because I care about my patients’ (Participant No. 15).

#### Hazards of insufficient knowledge

Although organizational learning has positive influences on preserving patient rights, nurses’ reluctance to gain modern knowledge and lack of learning culture in their organization are a threat to patient's lives. Therefore, being reluctant to the right of receiving favorable care can bring forth irreparable consequences. One nurse stated a significant event caused by him because of insufficient knowledge: ‘Our lack of knowledge can cost us the life of a patient. For example, when a patient is going to go the operation room, he needs to take heparin. I didn't know he should be on hold 4–6 h before. During operation, the patient was bleeding. It took a lot of effort to stop bleeding. It was possible for him to die’ (Participant No. 11).

### Informing the patients

One of the concerns that people have when they face health problems and diseases is the lack of knowledge. However, providing information about the disease and obtaining informed consent for making decisions about the appropriate treatment have been stipulated in the patients’ bill of rights. The results showed that having long-term contact with patients and being aware of their suffering caused nurses to prioritize informing patients and played an effective role in relieving patients’ concerns and satisfying them by trying to learn and improve their knowledge and skills.

Patients’ ignorance and fear of the unknown, resulting from failure to provide enough information to patients and lack of attention to informed consent, influenced the selection of an appropriate treatment by patients and the quality of their lives. One of the nurses in the dialysis unit quoted, ‘We have a patient who's been on dialysis for 17 years but has not yet received a transplant. He now has hyperparathyroidism and bone problems as well. He says he's afraid. Why? Because he's seen two or three patients who died after transplant. Also, the patient next to his bed was very bad after transplant surgery. Unfortunately, accurate and complete information is not given to patients. Good and bad points of each treatment are not mentioned to patients’ (Participant No. 3).

However, nurses’ efforts to acquire knowledge and skills caused patients to feel calm and relieved them from concerns. For example, a nurse said, ‘We had a patient who thought all his blood would be drained in the first session of dialysis and that he would be given new blood. He was crying his heart out, and then his blood pressure dropped. The patient was shocked. When I sat beside him and explained to him, he became very relaxed and accepted his disease. This surprised the rest of the patients’ (Participant No. 3).

Nurses designed training methods on the basis of the patients’ needs; as a result, they received good outcomes. ‘We saw that our patients have specific nutritional diets. We orally explained to them, but they kept forgetting it. Therefore, all contraindicated or restricted foods were written on a large colored poster, and it was posted in the waiting room. Patients can look at this poster when they are waiting to enter the doctor's room. When we examined their feedback, we found they were very satisfied’ (Participant No. 8).

However, there were obstacles that undermined the effectiveness of patient education by nurses. Lack of trust in nurses’ knowledge and lack of teamwork are examples of these obstacles. One of the nurses said, ‘This is a fact that what I, as a nurse, explain to patients is not as effective as what a doctor explains. This is our problem. I explain everything to my patients. But when the doctor comes, patients expect the doctor to confirm what I have told them. This is a reality that exists in our society. We don't train as a team. Doctors say their views and mostly the good prospects of the treatment. They don't say the complications and problems. This is why patients cannot decide’ (Participant No. 15).

Feeling satisfied with the services provided was one of the consequences of informing patients. One of the nurses said, ‘Once we had a patient with cholecystitis who was hospitalized several times. Finally, he was scheduled to undergo surgery, but the patient didn't consent to surgery. Later, that patient came here and said, ‘I didn't know that apart from surgical operation, there is laparoscopic method. When I was explained, and I selected surgery, I felt quite satisfied’ (Participant No. 13).

## Discussion

The experiences of the nurses who participated in this study showed that organizational learning had a positive role in respecting patient rights. It became feasible through motivating nurses to preserve patient safety through exploring and correcting errors, providing favorable care, being advocates, and informing the patients.

One of the major themes in this study was to preserve patient safety through exploring and correcting errors. Organizational learning is the ability of the organization as a whole to explore and improve errors (25), and the necessity to learn from the errors is emphasized because errors are opportunities to learn (26). The results of this study show that nurses perform a proactive risk assessment by being alert to the errors and implementing problem-identification processes. This approach allows nurses to learn all types of errors, factors involved in them, and the methods for their recovery (27), thus making the dream of organizational learning come true. The strategies being used by the nurses in this study are consistent with some earlier studies (28, 29). By creating teams inside the units, nurses provide an appropriate environment to think collaboratively and find the root causes of the problems. Such an environment is a way to organizational learning because learning starts in an organization when the teams start learning (26). The results of such trying were to prevent hurting the patient and preserve his/her safety. Organizational silence, in which people show the least reactions to answering major problems the organization faces, can threaten the life of the patient, which is just the opposite of the above mentioned approach (30). Considering the high prevalence of medical errors in hospitalized patients (31, 32), and the critical role of the nurses in preventing such errors, identifying, and improving them (33), it seems necessary to have a supportive environment to continue such behaviors. In such an environment, their effective role in preserving patient safety can be crystallized in all levels of the organization.

Another major theme taken from the analysis of the interviews was providing favorable care. This is a major concern in the world, and achieving a favorable quality for offering care is one of the major consequences of organizational learning. In the present study, nurses offered scientific, economical, and inexpensive suggestions that, after transferring information and through team work, could decrease or eliminate the risks and provide favorable care for the patient. This procedure includes colleagues’ learning and supporting the team members. Memarian et al. (34) and Motamed-Jahromi et al. (35) also showed similar results.

Nevertheless, nurses tried to offer the optimum care, even at their own personal costs. Though it causes care to be continued, encouraging nurses to be personally responsible for solving the existing problems becomes a burden to organizational learning, because in such situations, the opportunity for organizational promotion and applying changes decreases.

Nurses’ offering better care leads to patient trust and satisfaction. The importance of patient satisfaction is so emphasized that it is mentioned as a cornerstone in Iran's Standard guidelines. On the other hand, patient satisfaction has a positive and meaningful relationship with nurses’ job satisfaction (36, 37). Therefore, organizational learning, through having positive influence on the quality of the offered care, provides patient satisfaction in instances where patients are less satisfied with the services offered by the nurses (38, 39).

Being an advocate to the patient was another experience expressed by the participants. The nurses emphasized the patients’ protection, improvement, and continuation of care by expressing experiences concerning learning for favorable care and being alert about the risks resulting from nurses’ insufficient knowledge. These results are consistent with the studies by Mohadesh Motamed-Jahromi et al. (35), Barret-Sheridan (40), Negarandeh et al. (41), Henneman et al. (29), and Acebedo-Urdiales et al. (42). In these studies, nurses emphasized the nurses’ role of being advocates and described a positive attitude regarding this role. They defined being an advocate in a similar way as defined in the present study. More interestingly, the results of a study by Joolaee and Mehrdad showed that both patients and nurses have a common perception about patient rights (39). Despite the abovementioned, factors such as lack of power, lack of time, limited relationships (41), dominance of blaming culture, lack of skill and knowledge, and wrong management (43) prevented nurses from being advocates. In a study by Black, one-third of the nurses reported that though they were aware of the threatening conditions, they did not report it because they were afraid of its consequences (44). In a study by Bayazidi, half of the nurses who committed errors did not report them (45). Reporting errors and patient's risky conditions is one of the patient's protection dimensions, which is nowadays highlighted. However, lack of nurses’ skills and knowledge is a burden to handling threatening conditions and nurses’ being advocates. Therefore, it seems vital for the organization to lean toward organizational learning through governing an appropriate atmosphere for learning from errors, and their appropriate handling paves the way for patients to enjoy their rights of receiving favorable care and being protected against threats.

One of the primary themes of the interviews was informing the patients. When nurses expressed their experiences, it was found that they ignored patient rights to receive information about the disease and their treatment, and accordingly, they were unable to select an appropriate treatment, so that this issue could cause irreparable complications for the patient. This issue has also been stated in the study by Kirane et al. (46). According to their study, it was reported that only a quarter of the patients had received appropriate description about the procedures and their complications. However, providing information to patients and giving them the right to decide lead to satisfaction with the services provided. Few relevant studies have also reported the important role of informing patients in feeling satisfied with the services provided (47). However, obstacles, such as lack of trust in nurses and lack of teamwork, reduced the effectiveness of this training. Che et al. also pointed out these obstacles in their study (48). The results of this study emphasize the process of obtaining informed consent from patients and promoting teamwork in clinical wards.

## Conclusions

The results showed the fact that despite being disturbed by the health system's unkindness, nurses had no concern but offered favorable care and preserved patient safety. Therefore, it would be encouraging if nursing policymakers and managers appreciate nurses’ efforts to preserve patient safety and explore and improve errors. They are expected to highlight the role of nurses in patient satisfaction, to introduce them as patient advocates, and to appreciate their efforts for internalizing organizational learning.

## Paper context

High prevalence of medical errors, organizational silence, and patient dissatisfaction are the issues which indicate the negligence of patient rights. Furthermore, there is some evidence of nurses’ efforts to preserve patient safety and provide favorable care. Such an approach is accordant with organizational learning, which is expected to have a positive influence on these issues. The concept of organizational learning is not provided directly in undergraduate nursing degree curriculum. However, if it is introduced to nurses by measures, it can facilitate access to new knowledge and better performance along with organizational objectives for promotion and survival. Iranian hospitals, where clinical nurses work, have some individual and organizational factors that encourage nurses’ attempts toward acquiring knowledge and individual and organizational learning. These factors include facilitating access to knowledge resources by the organization, effective peer and team learning, tendency to individual development, and the need to be confirmed by doctors and other colleagues. This means nurses indirectly were faced with the concept of organizational learning and experienced its advantages practically. However, there is no clear picture of the relationship between these two concepts in literature. Therefore, conducting a qualitative study with an in-depth analysis is necessary.
